# Stereotactic Radiotherapy with Fractionation for the Lesions in and Around the Brainstem and Optic Nerve

**DOI:** 10.7759/cureus.6087

**Published:** 2019-11-06

**Authors:** Yoshihisa Kida, Yoshimasa Mori

**Affiliations:** 1 Neurosurgery, Ookuma Hospital, Nagoya, JPN; 2 Radiation Oncology and Neurosurgery, Center for Advanced Image-guided Radiation Therapy, Shin-Yurigaoka General Hospital, Kawasaki, JPN

**Keywords:** bed, brainstem, optic nerve, srs, srt

## Abstract

Purpose

Among the components of the central nervous system, the optic nerve and the brainstem are considered to be the eloquent structures that are sensitive to stereotactic radiosurgery (SRS) and stereotactic radiotherapy (SRT). SRS or SRT with fractionation in areas adjacent to these tissues is both promising and challenging.

Materials and methods

To clarify the precise dose distribution achievable with fractionation in and around the optic nerve and brainstem, theoretical simulations were performed, based on the biological effective dose (BED).

Results

These simulations clearly showed that the doses to the optic nerve and brainstem can be adjusted using fractionation, meaning that the prescribed doses to the surrounding brain tissue can be reduced. Conversely doses to the lesions themselves can be increased by fractionation, while maintaining a stable dose to normal optic nerve and brainstem tissue.

## Introduction

Radiosurgery has been established as one of the main treatment strategies for various brain lesions such as arteriovenous malformation (AVM), and both benign and malignant brain tumors as well as functional diseases such as trigeminal neuralgia, movement disorders and epilepsy. However, there are several obstacles to performing radiosurgery for the lesions in and around eloquent structures and for the lesions with large volumes. Some lesions in these locations can be treated with stereotactic radio surgery (SRS) or stereotactic radiotherapy (SRT). In terms of location, brain lesions in and around the optic nerve and brainstem are believed to be sensitive to the dose distribution. It seems to be extremely difficult to perform microsurgery without any troubles for the lesions in and around both locations. In this study, fractionated treatments for the lesions in and the around optic apparatus and brainstem will be estimated in order to obtain ideal dose distributions capable of excellent clinical results and no comorbidities. The most adequate and precise dose distributions are searched with simulation methods based on the biological effective dose (BED).

## Materials and methods

Current treatment results with radiosurgery

**Figure 1:**
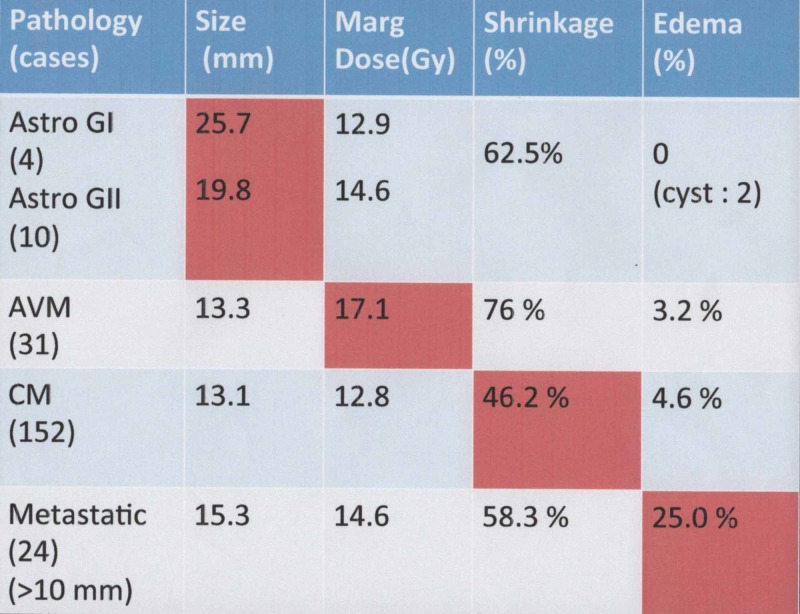
Current treatment results for lesions located in and around the optic nerve and brainstem. By using a single session of gamma knife radiosurgery, various lesions in and around optic nerve and brainstem have been treated at our institute.

Our current treatment results using radiosurgery for the lesions in and around the optic apparatus and brainstem are shown in Figure 1. For the brainstem lesions, marginal doses of between 12 to 15 Gy were currently chosen for the treatment of benign tumors. Brain metastases were treated with a mean marginal dose of 15.3 Gy. A higher marginal dose of 17.1 Gy was used for AVMs, while a lower dose of 13 Gy was selected for the treatment of symptomatic cavernous malformations. For optic gliomas, a dose distribution with less than 12 Gy at the margins was used because these lesions are not isolated from the optic apparatus. Therefore, assuming the doses of 15 Gy for brainstem lesions and 10 to 12 Gy for optic apparatus are seemed to be adequate and realistic. To optimize the dose distributions, simple radiosurgery procedure was compared with fractionated treatments. For further comparison, simulation study based on the BED, which reflects the different effects of radiation doses in different structures, was also examined.

Biological effective dose

**Figure 2:**
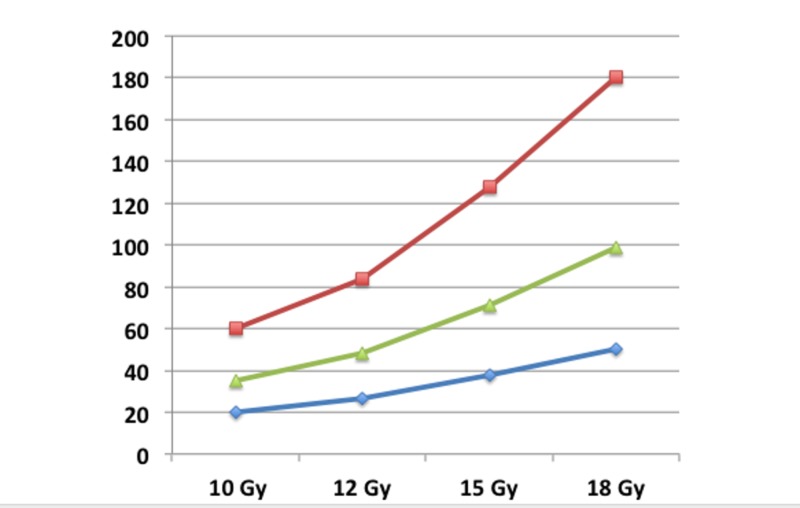
Relationships between increasing marginal doses and the biological effective dose of a single fraction. A delayed response is seen for normal brain tissue, and an acute response is seen for benign and malignant brain tumors. Red: normal brain; green: benign tumor; blue: malignant tumor; vertical bar: biological effective dose (GyE)

To evaluate the dose distribution inside the lesions and in their surrounding brain tissue, BED which is a widely accepted parameter, was utilized for comparisons. Currently the α/β ratios for normal brain, and benign and malignant brain tumors are assumed to be 2, 4, and 10, respectively. The relationship between the BED (GyE) and single irradiation is shown in Figure 2; this figure shows a sharp increase of the BED (GyE) in normal brain in association with an escalating single irradiation dose (Gy). Less remarkable increases in the BED were also confirmed for benign and malignant tumors as shown in Figure 2. A lessor increase of BED is revealed in benign and malignant tumors, indicating an apparent dissociation.

## Results

Relationship between BED and fractionation

Optic Nerve

When tumors in and around the optic apparatus are considered, a standard marginal dose of 12 Gy can be selected. To obtain the same BED as a single 12 Gy dose (85 GyE ), two to six fractionations of 8.2 Gy, 6.6 Gy, 5.5 Gy, 4.9 Gy and 4.4 Gy are required (Table 1).

**Table 1:** Estimated BEDs (GyE) in and around the optic nerve for between one and five fractions. While maintaining similar BEDs of the optic nerve by dose fractionations, those of benign and malignant tumors increase considerably. BED: Biological effective dose

	12 Gy x 1	8.2 Gy x 2	6.6 Gy x 3	5.5 Gy x 4	4.9 Gy x 5
BED (Optic N)	84	83.6	85.1	85.1	84.5
BED (Benign T)	48	50	52.5	53.8	54.5
BED (Malig T)	26.4	29.8	32.9	34.9	36.5
	11 Gy x 1	7.5 Gy x 2	6.0 Gy x 3	5.1 Gy x 4	4.45 Gy x 5
BED (Optic N)	71.5	71.3	72	72.4	71.7
BED (Benign T)	41.3	43.1	45	46.4	47
BED (Malig T)	23.1	26.3	28.8	30.8	32.2

**Figure 3:**
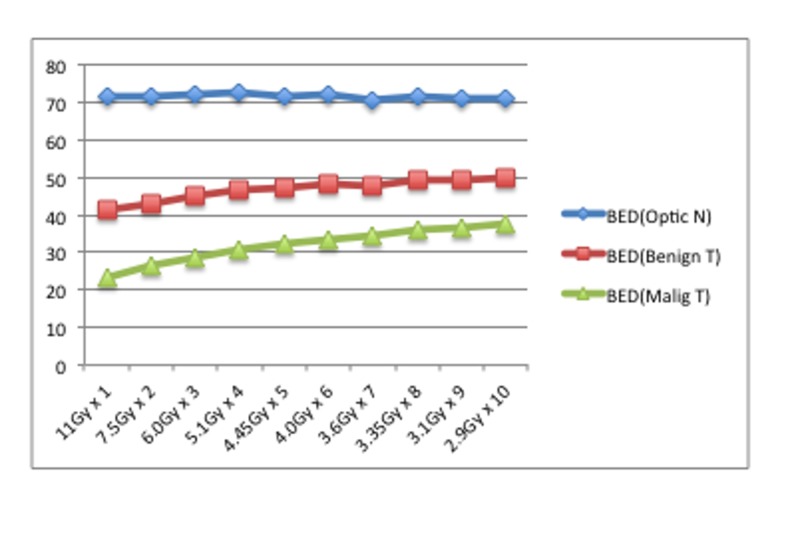
Theoretical relationships between single radiosurgery and the various fractionations. The BEDs (GyE) at the optic nerve were stable (blue), whereas the BEDs at benign (red) and malignant tumor (green) gradually increased with escalating fractionation. BED: Biological effective dose

In these situations, the BED for optic gliomas (α/β = 4) increases by almost 5 GyE in association with the more frequent fractionations. In malignant tumors (α/β = 10), the BED increases more sharply with more frequent fractionations (Figure 3).

**Figure 4:**
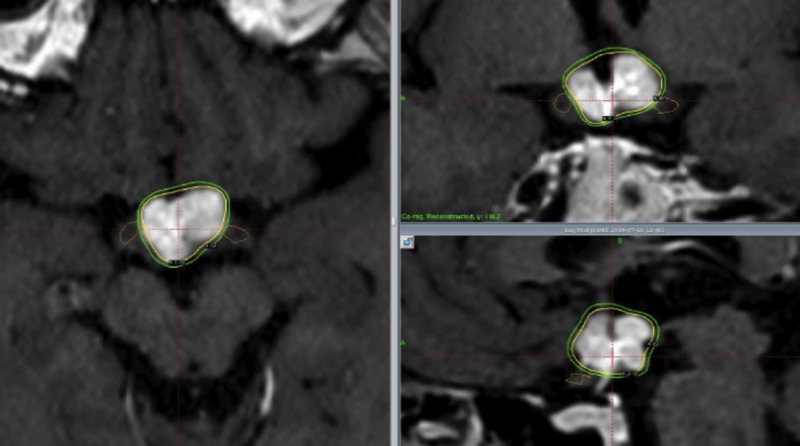
Hypothalamic glioma treated with fractionated gamma knife radiosurgery. Four fractions with 4.8 Gy (x 4 = 19.2 Gy) each were delivered to the lesion, which is almost equivalent as 11.2 Gy of single radiosurgery. Although the dose to the tumor is almost same, near-by optic nerves receive (brown) 10% lower dose than single radiosurgery.

Figure 4 shows the dose distribution for a hypothalamic glioma treated with fractionated radiotherapy.

A marginal dose of 4.8 Gy was delivered four times, but the BED was almost the same as that for a 11.2 Gy single fraction. In this situation, the nearby optic tracts received 9.3 Gy (Figure 4), which is equivalent to 52.5 GyE with BED.

Brainstem

Brainstem lesions can be treated using SRS with higher doses of 15 Gy at the margin by SRS. To maintain the similar BED (127.5 GyE), two to five fractions of 10.35 to 6.2 Gy are required (Table 2).

**Table 2:** Estimated BEDs in and around the brainstem for between one and five fractions. BED: Biological effective dose

	13 Gy x 1	8.9 Gy x 2	7.1 Gy x 3	6.05 Gy x 4	5.3 Gy x 5	% increase
BED (Brainstem)	97.5	97.91	96.9	97.4	98.3	0.8
BED (Benign T)	55.25	57.4	59.1	60.8	62.5	13.1
BED (Malig T)	29.9	33.6	36.4	38.8	41	33.8
	15 Gy x 1	10.05 Gy x 2	8.35 Gy x 3	7.05 Gy x 4	6.2 Gy x 5	% increase
BED (Brainstem)	127.5	127.3	128.2	127.6	127.1	0.5
BED (Benign T)	71.25	74.3	76.6	77.9	79.05	11.5
BED (Malig T)	37.5	42.12	45.6	48.1	50.2	33.9
	17 Gy x 1	11.75 Gy x 2	9.4 Gy x 3	8.1 Gy x 4	7.1 Gy x 5	% increase
BED (Brainstem)	161.5	161.5	160.7	163.6	161.5	0
BED (Benign T)	89.25	92.5	94.5	98	98.5	11.5
BED (Malig T)	45.9	51.1	54.7	58.6	60.8	32.3

**Figure 5:**
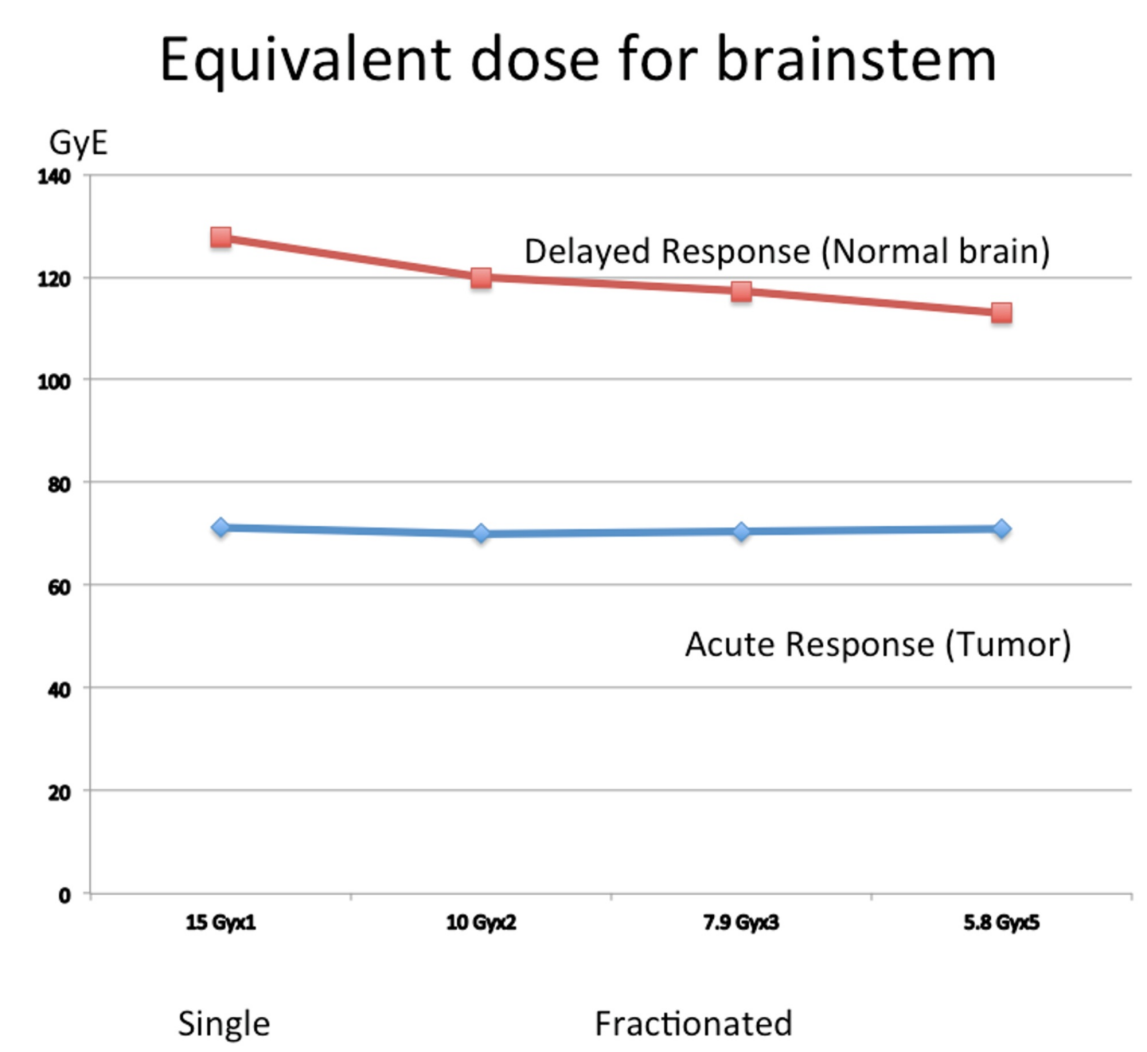
Equivalent dose for brainstem and tumors. When the BEDs are maintained stable in the tumor (blue) using fractionated procedures, the BEDs in normal brain tissue (red) can be reduced. BED: Biological effective dose

Conversely if fractionation is performed so as to maintain the same BED in the tumor, the BED in the normal brain tissue can be significantly reduced as shown in Figure 5.

**Figure 6:**
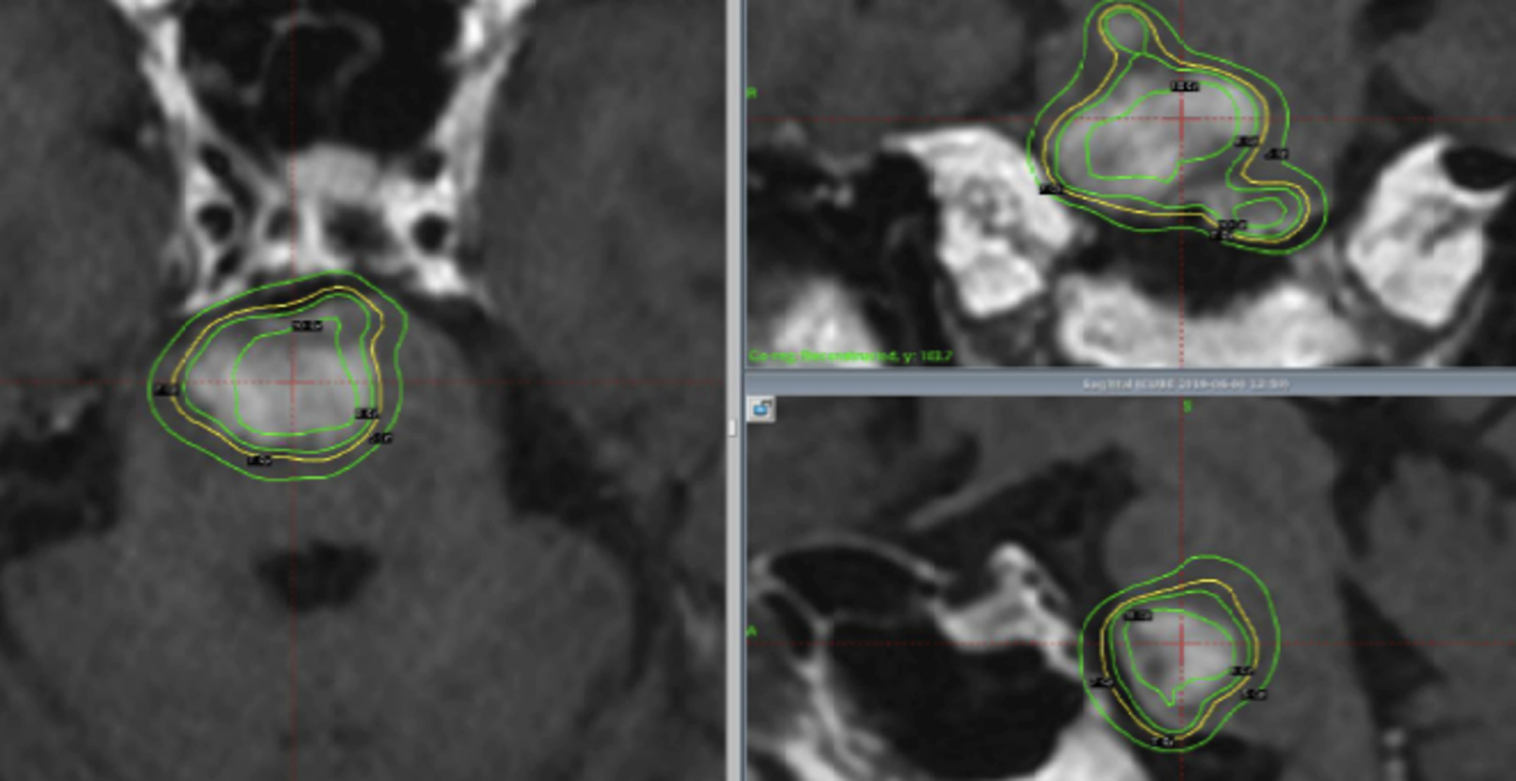
Pontine glioblastoma treated with fractionated gamma knife radiosurgery. Three fractions - 7.0, 6.7, and 6.7 Gy (20.4 Gy) - were delivered. The BED was almost equivalent to that received during single radiosurgery with 14.2 Gy at the margin. In this case, the surrounding brainstem received 15 GyE less than that of single radiosurgery. BED: Biological effective dose

A representative case of a pontine glioblastoma treated with three fractions is shown in Figure 6.

In this case, the surrounding brainstem received 15 GyE less than that of single radiosurgery.

**Figure 7:**
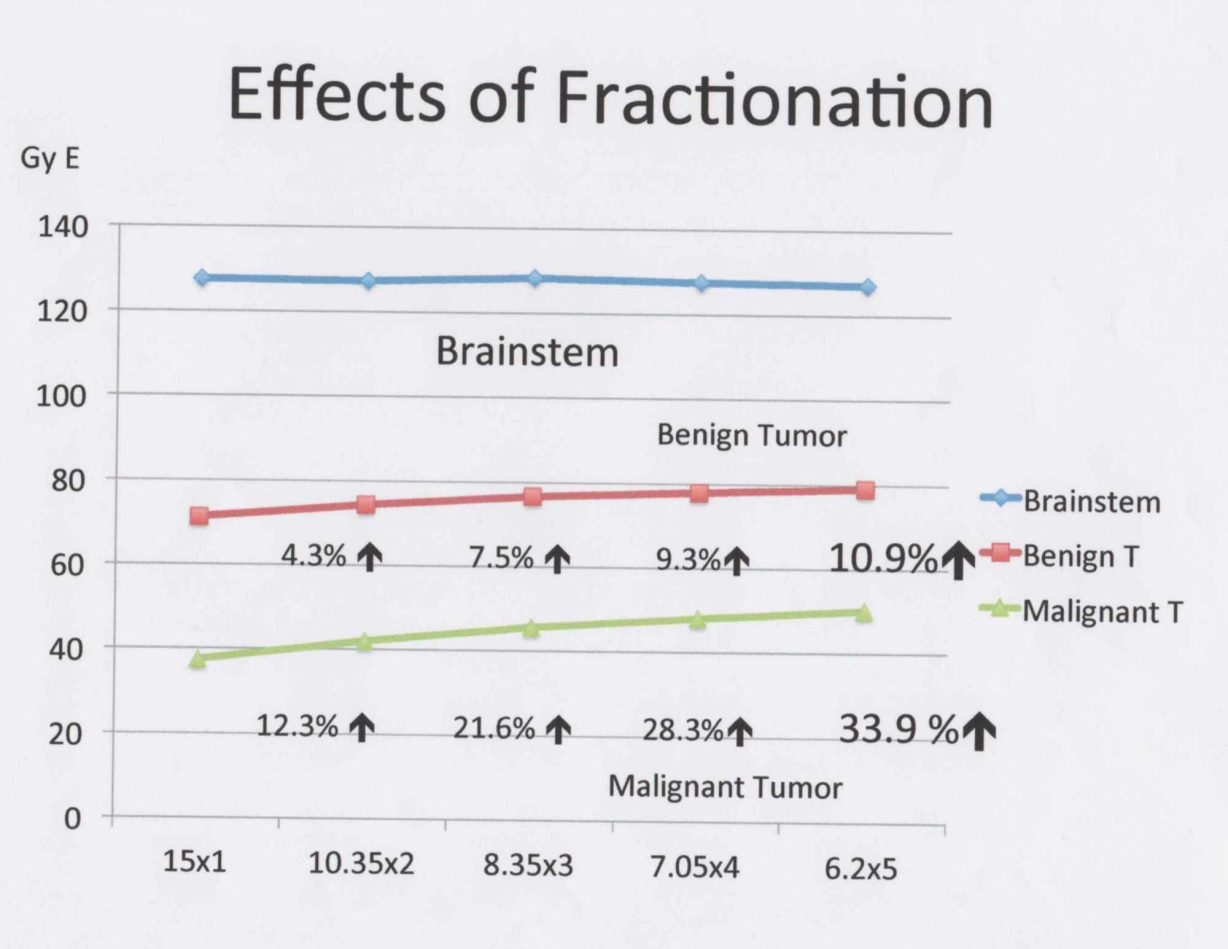
Using five fractionations, the BEDs in the tumors increased. While the BEDs in the brainstem remain stable, those in benign and malignant tumors can be increased by 10.9% and 33.9%, respectively, with fractionation. BED: Biological effective dose

In summary, if two to six fractions are assumed, the BED can be increased by approximately 10.9% for benign tumors and 33.9% for malignant tumors, compared with a single fraction (Figure 7).

## Discussion

Since the optic nerve and brainstem are very sensitive to irradiation, special care must be made when lesions in and around these structures are treated with SRT. Optic gliomas have been successfully treated with SRS in several reports [1-6]. The volumes of the treated tumors were not large and the marginal doses ranged between 10 and 13 Gy. The only main adverse effect was exclusively visual disturbance. The treatment of brainstem lesions with moderately high-dose SRS seems to be more difficult because of possible complications [7,8].

Adequate treatment methods for lesions in these two important structures have not yet been established. Therefore we performed a theoretical simulation to clarify the adequate dose distribution in these structures using the BED, which reflects the conversion of hypofractionated radiation doses to single doses using a linear quadrant (LQ) formula [9-11]. Selecting an adequate BED for radiation treatment, especially for SRT, is very helpful. However, the accuracy and validity of the BED, when it is applied to different tissues and structures, should be discussed, in addition to the effects of using different fractionation methods, such as different time intervals, and the use of various radiation sources and instruments.

There are various reasons for using fractionation. First, it is important to manage volume-dose relations and the burden to normal brain structures can be reduced using fractionation, since it allows recovery from sublethal brain damage. Second, the reoxygeneration process is accelerated during these intervals, and the sensitivity of the lesions itself to radiation may increase subsequently.

Optic nerve tumors or lesions

It is generally agreed that a maximum of 8 to 12 Gy can be delivered to the optic apparatus if visual acuity complications are to be avoided. In the present study, we clarified that adequate dose adjustments can be made using fractionation during stereotactic procedures. In fact, the marginal doses for the target can be increased considerably using two to five fractionations, while the doses to the optic apparatus can be kept stable and safe and comorbidities can be seemingly avoided. Conversely, the dose distributions for the target can be kept stable by fractionation, while the doses to the optic apparatus can be reduced. Various tumors can occur very near, but still separate from optic apparatus, including pituitary adenomas, craniopharyngiomas and parasellar meningiomas, those which are very near to optic nerve, but may have some distance. Since optic and hypothalamic gliomas exist inside the brain tissue, the marginal dose of the tumor is always adjacent or attached to the nerve. Moreover, the nerve fibers of the optic apparatus may penetrate the tumor in some situation. Fractionated radiosurgery or radiotherapy may be helpful in these cases, since BED to the nerve and tumor can be adjusted appropriately.

Brainstem

Small tumors in and around the brainstem can be treated safely using radiosurgery. Since the brainstem is the most eloquent structure in the central nervous system, the treatment of large tumors or lesion in the brainstem is difficult, because of potential for comorbidities or neurological deficits [12-15]. In the present study, we clarified that the dose to the target can be increased by fractionation in the same manner as for tumors in the vicinity of the optic apparatus. However, the marginal doses for the brainstem targets can be quite different in radiosurgery, depending on the pathology of the lesion. For example, brainstem cavernous malformations can be treated with a single radiosurgery dose of less than 13 Gy at the margin, whereas a marginal dose of almost 15 Gy or more is typically used in the radiosurgical treatment of metastatic tumors or AVMs. The use of fractionation is extremely important for preserving brainstem function as well as for avoiding complications. This is especially true in the treatment of large lesions located in or around brainstem. Since the doses in and around the brainstem can be adjusted, adequate fractionation should be considered for the treatment of large tumors and complicated lesions.

## Conclusions

A theoretical simulation study of SRS or SRT was performed to evaluate the treatment of tumors in and around the optic nerve and brainstem. We clarified that the radiation doses to both normal brain tissue and tumors can be adjusted using fractionated treatment. With the fractionation methods, large brainstem tumors and the tumors in most eloquent sites might be possible.
